# Cascade synthesis and optoelectronic applications of intermediate bandgap Cu_3_VSe_4_ nanosheets

**DOI:** 10.1038/s41598-020-78649-9

**Published:** 2020-12-10

**Authors:** Mimi Liu, Cheng-Yu Lai, Meng Zhang, Daniela R. Radu

**Affiliations:** 1 Department of Mechanical and Materials Engineering, Florida International University, Miami, FL 33174 USA; 2 School of Materials Science and Engineering, Georgia Institute of Technology, Atlanta, GA 30332 USA

**Keywords:** Materials for devices, Materials for energy and catalysis, Nanoscale materials

## Abstract

Two-dimensional (2D) ternary materials recently generated interest in optoelectronics and energy-related applications, alongside their binary counterparts. To date, only a few naturally occurring layered 2D ternary materials have been explored. The plethora of benefits owed to reduced dimensionality prompted exploration of expanding non-layered ternary chalcogenides into the 2D realm. This work presents a templating method that uses 2D transition metal dichalcogenides as initiators to be converted into the corresponding ternary chalcogenide upon addition of copper, via a solution-phase synthesis, conducted in high boiling point solvents. The process starts with preparation of VSe_2_ nanosheets, which are next converted into Cu_3_VSe_4_ sulvanite nanosheets (NSs) which retain the 2D geometry while presenting an X-ray diffraction pattern identical with the one for the bulk Cu_3_VSe_4_. Both the scanning electron microscopy and transmission microscopy electron microscopy show the presence of quasi-2D morphology. Recent studies of the sulfur-containing sulvanite Cu_3_VS_4_ highlight the presence of an intermediate bandgap, associated with enhanced photovoltaic (PV) performance. The Cu_3_VSe_4_ nanosheets reported herein exhibit multiple UV–Vis absorption peaks, related to the intermediate bandgaps similar to Cu_3_VS_4_ and Cu_3_VSe_4_ nanocrystals. To test the potential of Cu_3_VSe_4_ NSs as an absorber for solar photovoltaic devices, Cu_3_VSe_4_ NSs thin-films deposited on FTO were subjected to photoelectrochemical testing, showing p-type behavior and stable photocurrents of up to ~ 0.036 mA/cm^2^. The photocurrent shows a ninefold increase in comparison to reported performance of Cu_3_VSe_4_ nanocrystals. This proves that quasi-2D sulvanite nanosheets are amenable to thin-film deposition and could show superior PV performance in comparison to nanocrystal thin-films. The obtained electrical impedance spectroscopy signal of the Cu_3_VSe_4 _NSs-FTO based electrochemical cell fits an equivalent circuit with the circuit elements of solution resistance (R_s_), charge-transfer resistance (R_ct_), double-layer capacitance (C_dl_), and Warburg impedance (W). The estimated charge transfer resistance value of 300 Ω cm^2^ obtained from the Nyquist plot provides an insight into the rate of charge transfer on the electrode/electrolyte interface.

## Introduction

A major advantage of the 2D materials, whether intrinsically layered in structure or not, is their amenability to solution processing and deposition on flexible substrates. Ultra-thin films fabricated from 2D materials could survive sustained stress and strain compliance on flexible supports, and thus, could revolutionize diverse applications such as optoelectronics, valleytronics, energy harvesting, conversion and storage, and biomedicine^1–3^.

Among several classes of 2D compounds, transition metal dichalcogenides (TMDCs) gained considerable interest^4–6^ after graphene isolation^7^ in 2004. While graphene features a zero-band-gap, the plethora of 2D inorganic materials in the TMD family could act as insulators (e.g., hexagonal boron nitride–hBN), semiconductors (e.g., MoS_2_, WS_2_, WSe_2_), or superconductors (e.g., NbSe_2_, NbS_2_), on the account of their various crystal structures and polytypism. Structurally, TMDCs with the general formula of MX_2_, have an individual layer of transition metal atoms (M = Mo, W, Ta, etc.) sandwiched between two chalcogen layers (X = Se, S, Te)^8^. The plethora of metal–chalcogen combinations and the large number of synthetic methods to fabricate these useful materials make TMDCs a rich platform for further chemical transformations, and we aim to demonstrate synthetic methods to convert them into 2D ternary materials.

Recently, 2D ternary materials raised interest in optoelectronics and energy-related applications, given the degree of freedom added through introduction of the third element^9^. Four types of 2D ternary metal chalcogenides have been identified and synthesized in the past few years: 2D ternary chalcogenides with well-defined ternary layered crystal structures (e.g., TaNiS_5_ and Cu_2_WS_4_), alloyed TMD nanosheets (e.g., MoS_2x_Se_2(1−x)_ and Mo_x_W_1−x_S_2_, where x = 0.5), heteroatom-doped TMD nanosheets (e.g., Re-, Co-, V-, Cr-, or Pt-doped MoS_2_), and lateral or vertical metal chalcogenide hetero-nanostructures (e.g., MoS_2_–MoSe_2_ and MoS_2_–WS_2_)^10^.

Introduction of a heteroatom into a ultrathin 2D TMDC nanosheets to construct a 2D ternary metal chalcogenide nanosheet is a compelling way to obtain unprecedented morphologies of compounds known only in bulk^10^. Ternary 2D nanosheets are of recent interest in important applications, including energy storage (e.g. TiNb_2_O_7_—due to its high theoretical specific capacity)^11,12^; high-performance photodetectors (e.g. CuInSe_2_), and highly-sensitive/selective fluorescence DNA sensors (e.g. Ta_2_NiS_5_)^13^.

Various synthetic strategies have been explored to produce 2D ternary materials with well-defined ternary 2D crystal structures, comprising top-down exfoliation, bottom-up chemical vapor deposition (CVD), and solution synthesis methods^14^; often, a combination of two different methods, e.g., chemical vapor transport (CVT) and mechanical exfoliation, is required for preparation of the final product^9^. Several challenges, including harsh preparation conditions, and subsequently safety of the process, along with reproducibility and preparation time^9,10,13,15^ are encountered in all of the aforementioned approaches. To advance the field of 2D ternary materials and accommodate an increasing demand for a number of applications, versatile synthetic methods that are robust, simple, safe and time-effective, became a practical necessity. Solution-based methods have several significant advantages over solid-state methods to synthesize nanomaterials including: (1) low reaction temperatures; (2) size-selective growth; and (3) morphological control^16^.

The class of sulvanites Cu_3_MX_4_ (M = V, Nb, Ta; X = S, Se, Te), received recent attention for the outstanding optoelectronic properties and promising thermoelectric properties^17–20^, making these compounds attractive for application as solar photovoltaic absorbers, transparent conductors, ion conductors and photocatalysts^19,21,22^.

The ability to generate such materials in 2D could enable fabrication of ultra-thin-films that would revolutionize a variety of applications. However, typical one-pot solution-based synthesis utilized for synthesizing copper chalcogenides for many of these materials result in nanoparticles^23,24^.

The sulvanite family is known for decades, but it was only recently explored at the nanoscale. Solution-phase synthesized Cu_3_VS_4_ nanocrystals^23,25^ show an interesting UV–Vis absorption, with three absorption peaks in the visible range, attributed to the presence of the intermediate band gap (IB) in their electronic structure^25^. We recently showed that Cu_3_VSe_4_ nanocrystals, synthesized through a solution process, showcase a similar UV–Vis absorption pattern, suggesting the presence of the IB in the nanoscale vanadium-sulvanites^26^.

Intermediate band semiconductors recently raised special attention for their potential to exceed the Shockley–Queisser limits in thin-film solar photovoltaics. Semiconductors with an intermediate band can absorb energies below the bandgap energy through two optical transitions from the valence to the intermediate band and from the intermediate to the conduction band, resulting in enhanced conversion efficiency^27–29^. Besides, the three optical transitions caused by the intermediate band can also lower the energy losses relying on thermal relaxation of optically excited carriers^30^. Theoretical predictions for a solar cell using an intermediate band semiconductor as absorber, could reach an efficiency of 63.1% in a single junction, exceeding the Shockley–Queisser barrier^31^.

We recently demonstrated that kesterite Cu_2_ZnSn(S,Se)_4_ (CZTSSe) could be prepared in a quasi-2D morphology through a cascade reaction starting from a binary chalcogenide SnSe_2_ with nanosheet morphology. Upon addition of copper and subsequently zinc, quasi-2D CZTSSe with inherited nanosheet morphology formed^32^. Our recent report on synthesizing Cu_3_VSe_4_ cubic nanocrystals using a hot-injection method, paved the road toward obtaining nanoscale Cu_3_VSe_4_. The method involves temperatures up to 260 °C, and leads to a high purity material in matter of hours^26^, in comparison with the six weeks required by the solid state synthesis conducted at 600 °C^33^.

Our hypothesis was that Cu_3_VSe_4_ could be synthesized in nanosheet morphology, and that the approach is amenable to other multinary chalcogenides, including other sulvanites. In this work, we demonstrate a facile synthesis of Cu_3_VSe_4_ nanosheets (NSs) using the same template approach that allows a seamless morphing of the nanosheet from the binary compound to the ternary one. The solution-phase process involves synthesis of VSe_2_ nanosheets followed by insertion of Cu(II) cations in the same reaction vessel, leading to Cu_3_VSe_4_ nanosheets.

VSe_2_ nanosheets were prepared at 250 ℃ by hot injection synthesis and characterized by X-ray diffraction and transmission electron microscopy (TEM), demonstrating the 2D morphology. Upon introducing Cu cations into VSe_2_ nanosheets, the quasi-2D Cu_3_VSe_4_ formed, exhibiting large surface area (micron size) nanosheets, thus retaining the template morphology. The Cu_3_VSe_4_ formation mechanism was investigated by X-ray diffraction, indicating that the insertion and new compound formation happens within minutes. It is remarkable that the Cu_3_VSe_4_ nanosheets maintain the crystal structure of bulk sulvanites (cubic), as identified by XRD. The 2D morphology was confirmed by TEM and SEM. Three distinct absorption bands similar to Cu_3_VS_4_ NCs were investigated by UV–Vis–NIR measurements. Additionally, the photocurrent generated by a Cu_3_VSe_4_ NSs thin film on FTO evidences the potential of Cu_3_VSe_4_ NSs in solar photovoltaic applications.

## Results and discussion

### VSe_2_ nanosheets characterization

**Figure 1:**
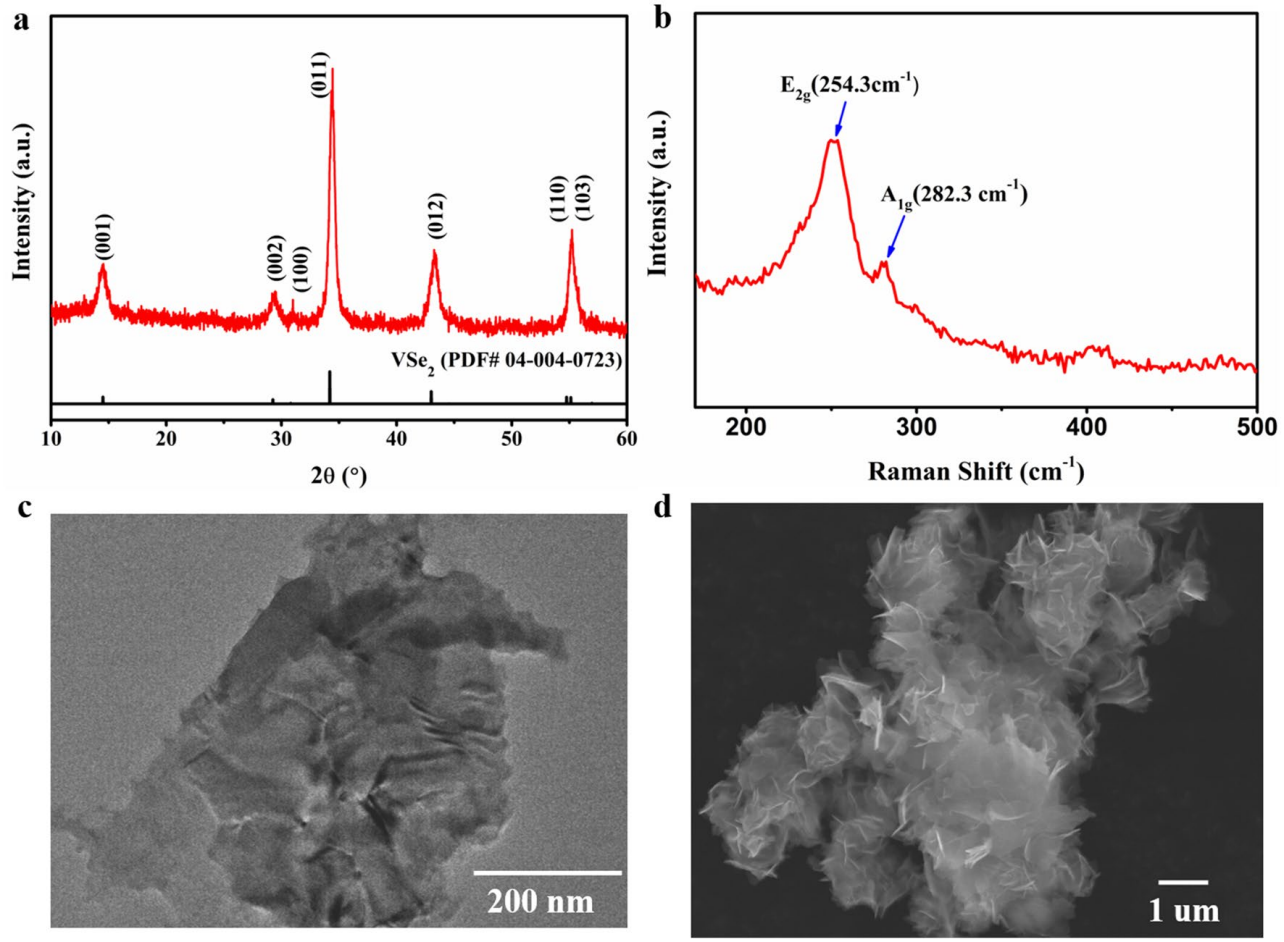
Characterization of the synthesized VSe_2_ NSs. (**a**) X-ray diffraction. (**b**) Raman spectrum. (**c**) TEM image of a nanosheet. (**d**) SEM image of a nanosheet agglomerate.

The VSe_2_ nanosheets, obtained through a modified literature procedure^34^, show high crystallinity; the X-ray diffraction pattern of the synthesized VSe_2_ corresponds to the 1 T-VSe_2_ polytype^35^ belonging to the *P* ¯3*m*1 space group (PDF# 40723) (Fig. 1a). The Raman spectrum of the synthesized VSe_2_ (Fig. 1b) exhibits two primary peaks (254.3 cm^−1^ and 282.3 cm^−1^) where the primary peak (around 251.3 cm^−1^) corresponds to E_2g_ in-plane vibration mode while the peak at 286 cm^−1^ is consistent with A_1g_ out-of-plane vibrational mode. The slight redshift (5–10 cm^−1^) when compared with literature reported data could be attributed to the layered structure^34,36^. The TEM image in Fig. 1c shows micrometer-size lateral dimension VSe_2_ NSs. The SEM image of the synthesized VSe_2_ nanosheets (Fig. 1d), further confirms the nanosheet morphology. The powder subjected to SEM was not subjected to exfoliation and shows an aggregate of VSe_2_ nanosheets.

### Cu_3_VSe_4_ nanosheets characterization

The preparation of Cu_3_VSe_4_ NSs presented herein demonstrates the ability to maintain the morphology and the large dimension of the template upon copper introduction into VSe_2_ NSs. By contrast, a similar copper insertion, done at 25 °C, was reported for In_2_Se_3_; however, the experiment rendered wrinkled CuInSe_2_ nanosheets which did not resemble the template. Further, the same reaction conducted at elevated temperatures results in nanodisks^37^.

**Figure 2:**
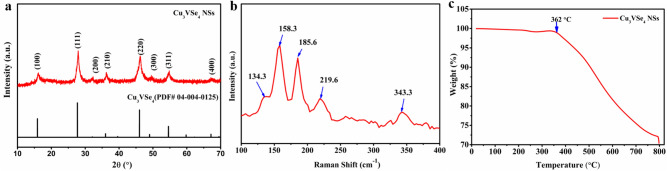
Characterization of Cu_3_VSe_4_ NSs. (**a**) XRD pattern. (**b**) Raman spectrum. (**c**) TGA plot.

The X-ray diffraction pattern in Fig. 2a, agrees with the bulk XRD, showing cubic Cu_3_VSe_4_ structure (PDF# 40125, a = b = c = 5.572 Å) in the *P* ¯43*m* space group. Raman spectra of as-synthesized Cu_3_VSe_4_ in Fig. 2b presents five peaks (134.3 cm^−1^, 158.3 cm^−1^, 185.6 cm^−1^, 219.6 cm^−1^, and 343.3 cm^−1^), consistent with the Cu_3_VSe_4_ nanocrystals Raman^26^. The intensity ratio of the peaks of Cu_3_VSe_4_ NSs is different from the ones attributed to the nanocrystals could be attributed to the morphology change^34,38^.

To investigate the thermal stability of the Cu_3_VSe_4_ nanosheets, thermogravimetric analysis (TGA) was performed in the temperature interval 25–800 ℃ at a ramping rate of 20 ℃ min^−1^, under argon atmosphere. Figure 2c displays the TGA curve of the synthesized Cu_3_VSe_4_ nanosheets, showing a rapid weight loss that starts at 362 ℃, indicating the decomposition onset. As expected, Cu_3_VSe_4_ with nanosheet morphology have much lower thermal stability than Cu_3_VSe_4_ nanocrystals. The slight weight loss between 200 and 350 ℃ is characteristic to the decomposition of the organic ligand residues originated from the synthesis.

**Figure 3:**
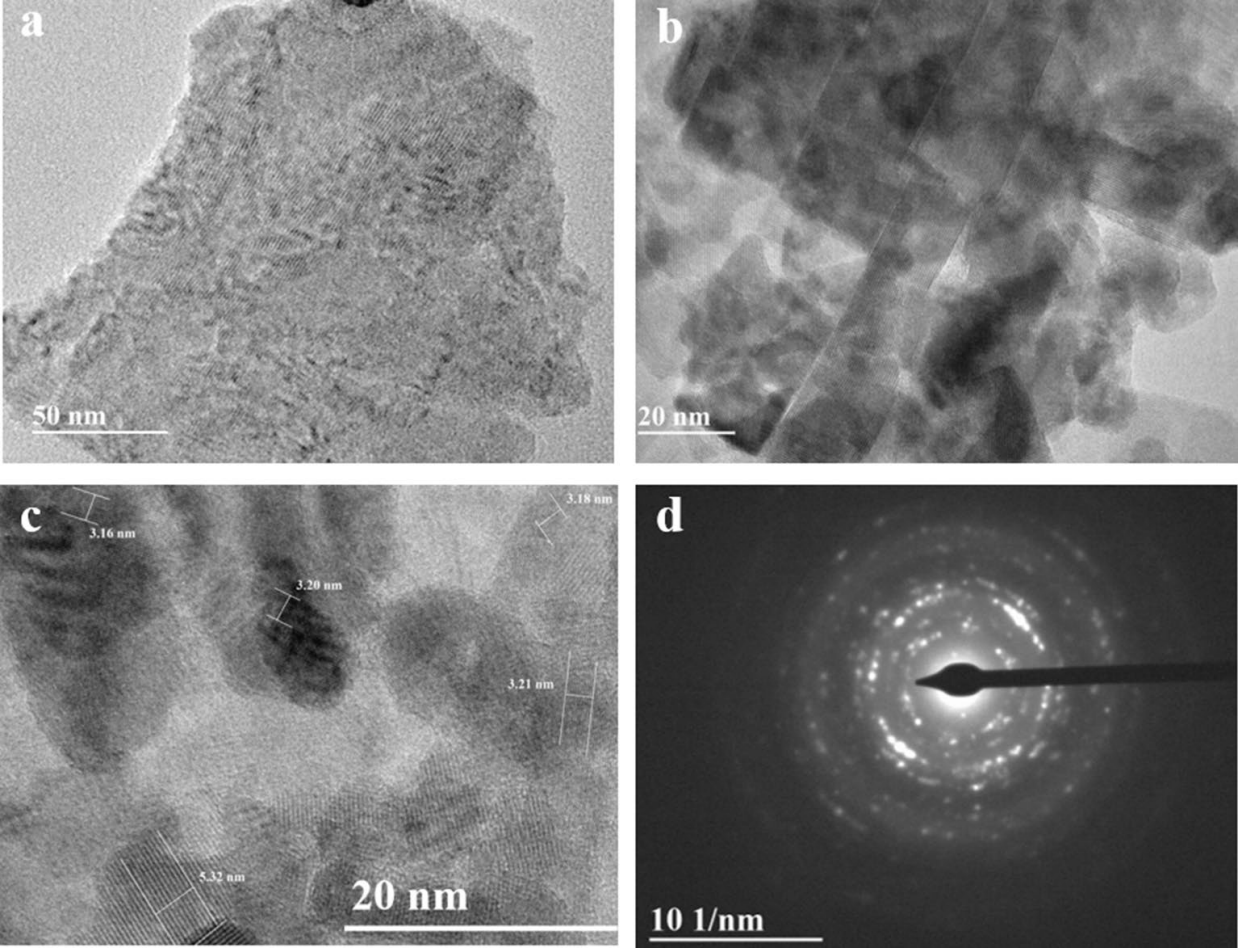
(**a**) Low-resolution TEM image. (**b**) TEM image. (**c**) HRTEM image. (**d**) SAED pattern of synthesized Cu_3_VSe_4_ nanosheets.

**Figure 4:**
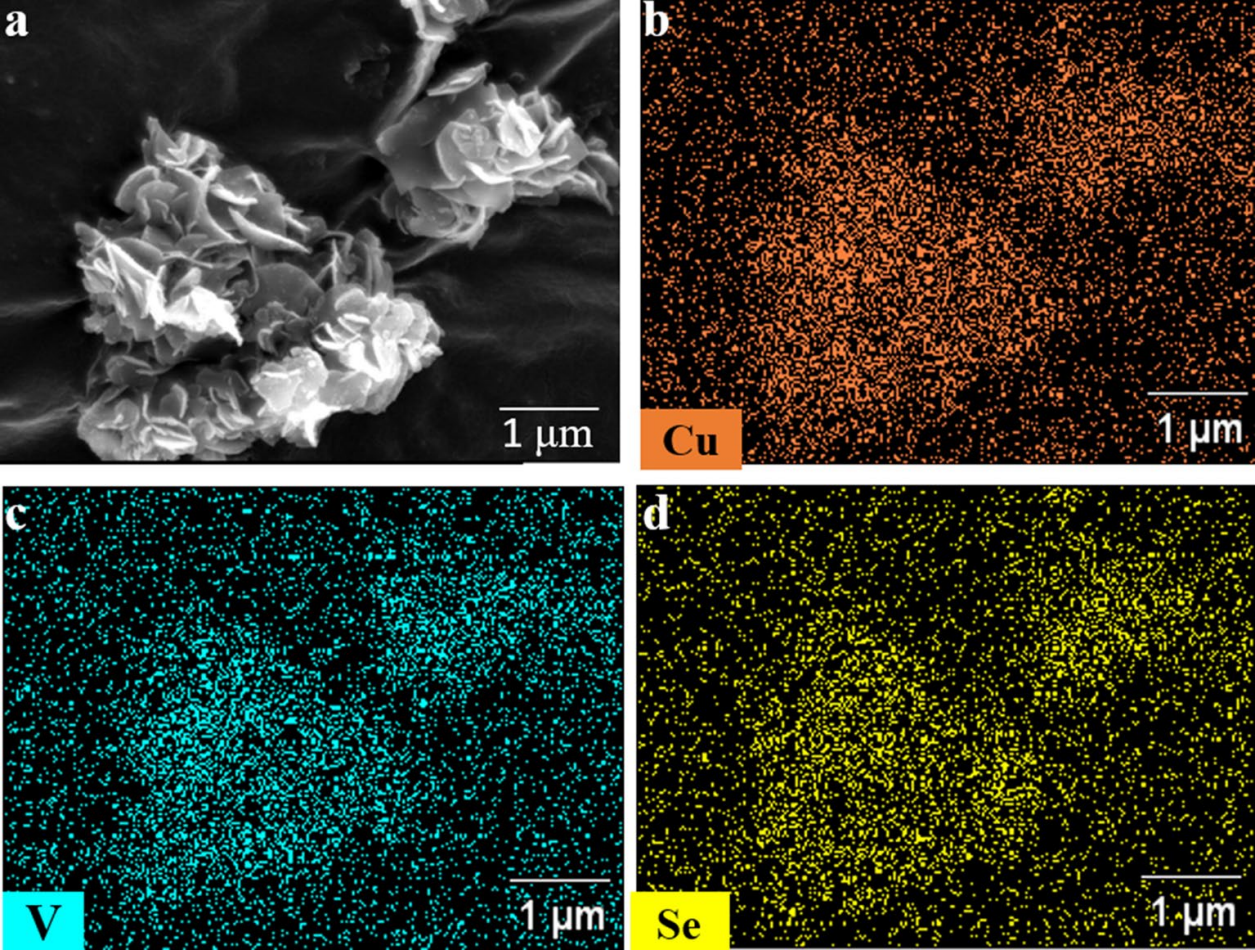
(**a**) SEM images of synthesized Cu_3_VSe_4_ nanosheets. EDS elemental mapping of (**b**) Cu, (**c**) V, and (**d**) Se.

**Figure 5:**
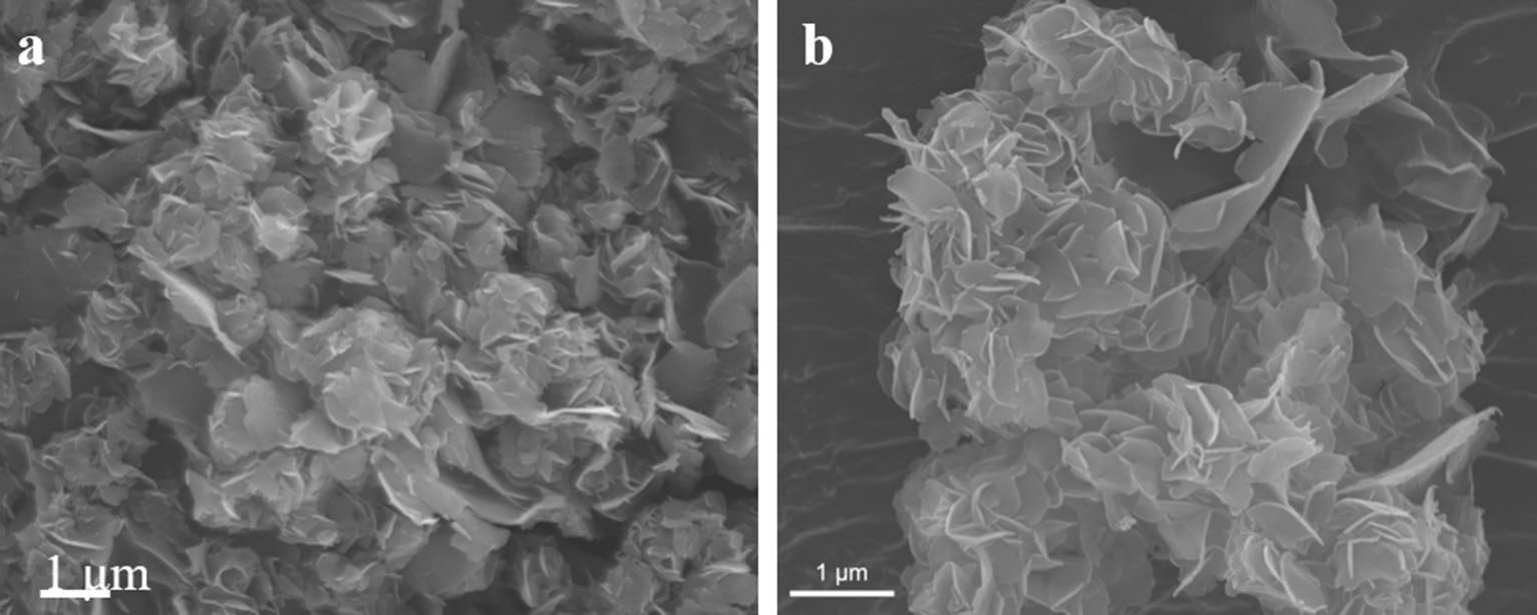
SEM image of (**a**) starting material VSe_2_ NSs. (**b**) Final product Cu_3_VSe_4_ NSs.

The morphology of the Cu_3_VSe_4_ NSs was evaluated by TEM and SEM. At low magnification, TEM imaging (Fig. 3a) reveal a nanosheet morphology with Cu_3_VSe_4_ NSs possessing a large lateral dimension (several hundred nanometers). At higher magnification (Fig. 3b) the images suggest a belt-shape concatenation of small a square-shaped nanocrystal, which self-assemble to render the quasi-2D nanosheets. The d-spacing of ~ 5.32 nm and 3.21 nm, determined by electron diffraction (Fig. 3c,d) correspond to the (100) and (111) facets of cubic Cu_3_VSe_4_. The nanosheet morphology is further supported by SEM imaging (Figs. 4a and 5); the nanosheets self-assembly in an arrangement reminiscent of flower petals. The uniform distributions of Cu, V, Se is reflected in the elemental maps collected by SEM–EDS (Fig. 4b–d, respectively). Importantly, the thickness of the nanosheets subjected to AFM is in the vicinity of 10 nm, as illustrated in Figure S1 (“Supporting Information”). As shown in Fig. 5, using VSe_2_ NSs as template, we obtained Cu_3_VSe_4_ NSs, demonstrating the ability of the synthetic method to maintain the morphology and the large dimension of the starting materials. To assess the chemical and electronic structures of the synthesized Cu_3_VSe_4_ nanosheets, XPS was performed as shown in Supplementary Figure S2, indicating that the oxidation states of Cu and Se in Cu_3_VSe_4_ NSs are + 1 and − 2, respectively, as the same in the Cu_3_VSe_4_ nanocrystals, and the vanadium is in presence of V^5+^ but with a negative shift of 2.5 eV in the binding energy of V^5+^ (The detailed discussion can be found in “Supporting Information”)^26^.

### Study of the Cu_3_VSe_4_ formation mechanism

**Figure 6:**
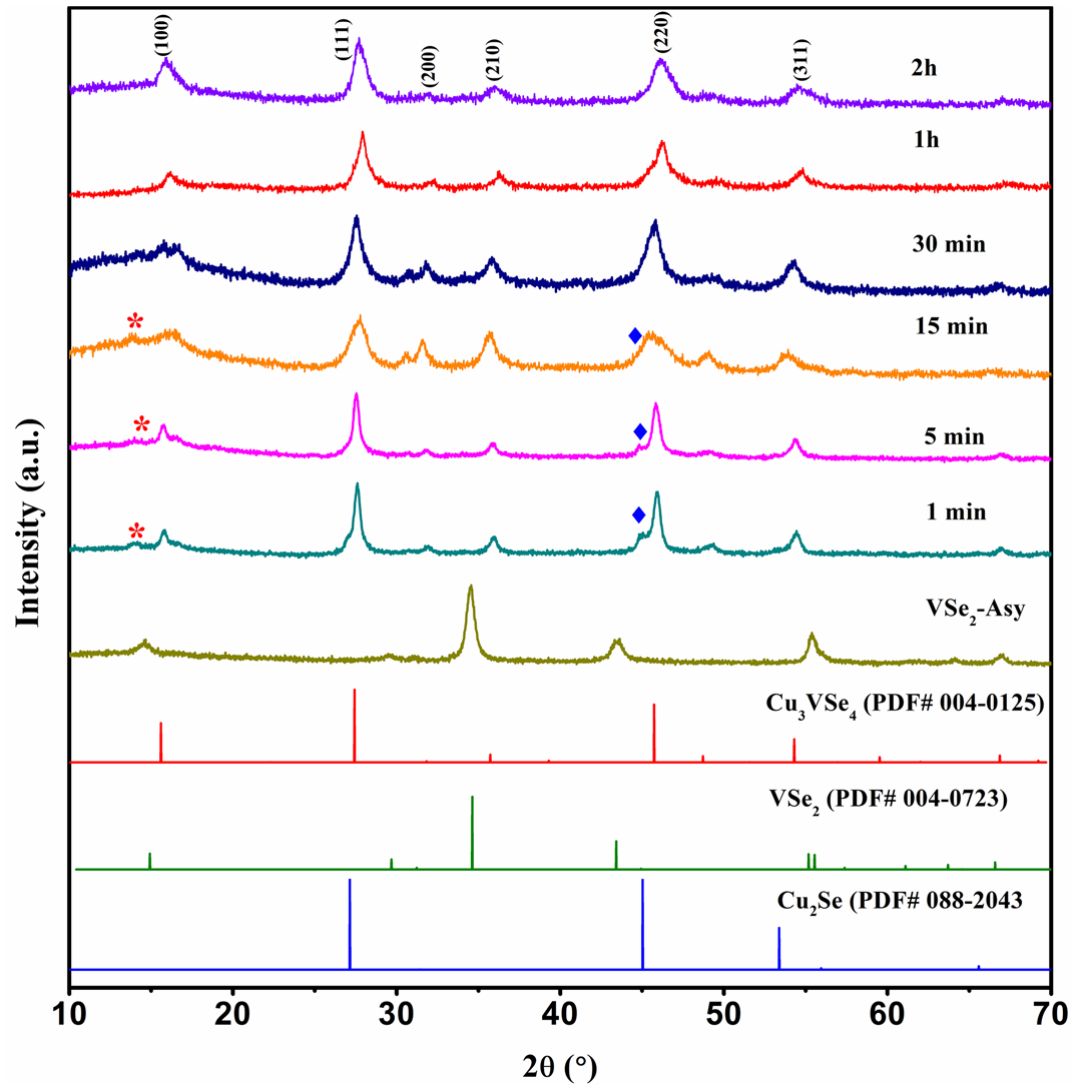
XRD patterns of Cu_3_VSe_4_ product using different reaction time.

To get insight into the conversion mechanism of VSe_2_ NSs to Cu_3_VSe_4_ NSs, we conducted a time study, involving a series of syntheses with all parameters kept constant, excepting the reaction times. The corresponding XRD patterns of the products are showed in Fig. 6. The study indicated that the primary crystalline phase in all products is cubic Cu_3_VSe_4_ with representative 2θ peaks of 15.9°, 27.7°, and 46.03°. Interestingly, Cu_3_VSe_4_ NSs forms within 1 min after the Cu^2+^ injection. A minor amount of Cu_2_Se and trace amounts of VSe_2_ were present in the products for the reactions conducted for 1 min, 5 min, and 15 min. When the reaction time exceeded an hour, VSe_2_ templating material and Cu_2_Se impurities are no longer present and the sole product was Cu_3_VSe_4_ NSs. Likewise, when extending the reaction time to two hours, the product shows the same Cu_3_VSe_4_ phase with a slightly higher crystallinity. It is important to note that no decomposition occurs.

We posit that the 2D layering nature of the VSe_2_ template provides a short diffusion distance for the injected Cu^2+^ which, along with the large reaction surface area, enables a short reaction time. Provided that the reaction is conducted in a cascade, involving VSe_2_ formation and further addition of Cu(II), unreacted Se from VSe_2_ formation could react with Cu(II) resulting in Cu_2_Se impurities. It is conceivable that as the reaction proceeds, the formed Cu_2_Se and unreacted VSe_2_ NSs form Cu_3_VSe_4_. In all reactions, the formed Cu_3_VSe_4_ maintains the 2D morphology.

### Optical properties of Cu_3_VSe_4_ NSs

**Figure 7:**
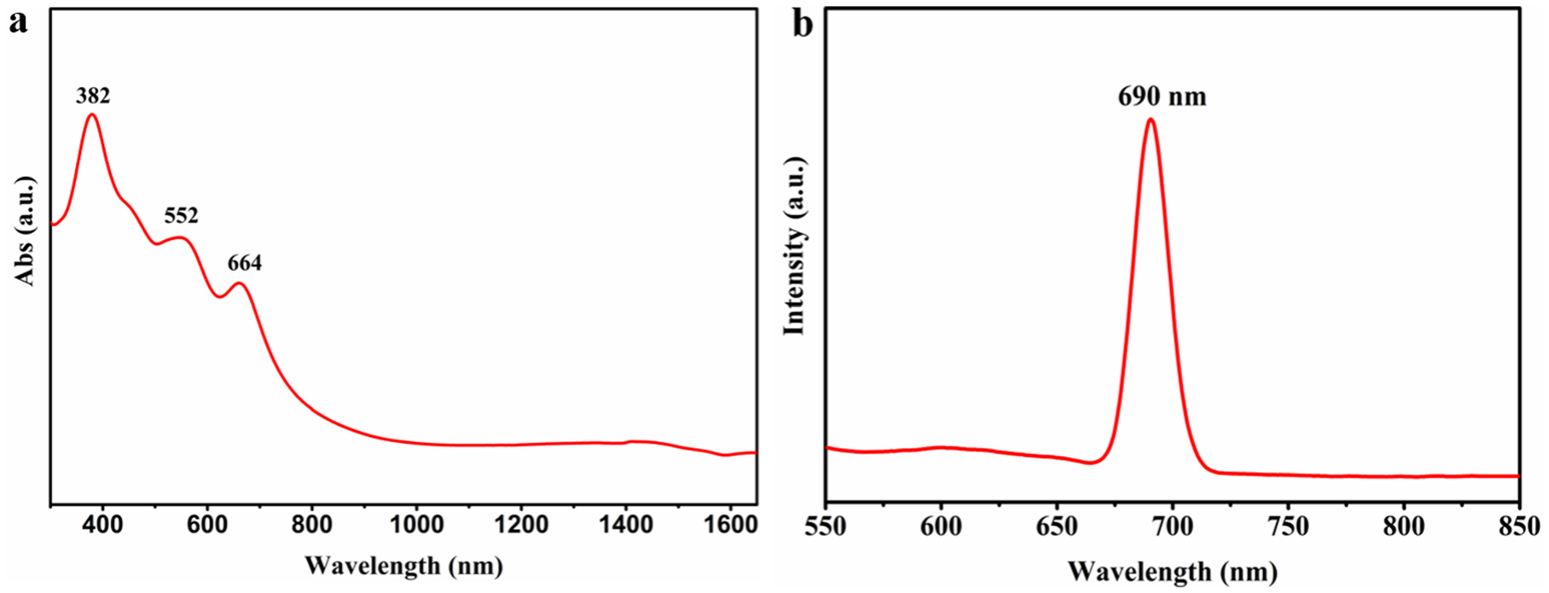
(**a**) UV–Vis–NIR spectrum. (**b**) Photoluminescence (PL) spectrum of the synthesized Cu_3_VSe_4_ NSs in ethanol.

To assess absorption characteristics of Cu_3_VSe_4_ nanosheets, UV–Vis–NIR spectra of the synthesized Cu_3_VSe_4_ NSs were measured (Fig. 7a; all measurements used Cu_3_VSe_4_ NSs ethanol dispersions). All spectra show three absorption peaks located at around 382 nm, 552 nm, and 664 nm. The three peaks are a signature of the nanoscale sulvanites studied to date (Cu_3_VSe_4_ and Cu_3_VSe_4_ NCs) and it could be inferred from all published work on several ternary chalcogenides that the Cu_3_VSe_4_ with the nanosheet morphology present an intermediate bandgap. Similar to reported assignments for Cu_3_VS_4_ and Cu_3_VSe_4_ NCs, and other ternary chalcogenides^26,27,39^ the three absorption peaks could be ascribed to the following bandgaps: VB-CB (3.24 eV), VB-IB I (2.24 eV), and VB-IB II (1.86 eV), respectively, when converting wavelength in photon energy.

Photoluminescence (PL) measurements were conducted to determine the bandgap of the nanosheets for comparison with the reported theoretical bandgap. Figure 7b shows the photoluminescence (PL) spectrum of as-synthesized Cu_3_VSe_4_ NSs, with a significant emission peak at 690 nm when using 460 nm as the excitation wavelength. Interestingly, the variation of the excitation wavelength from 430 to 520 nm at 10 nm intervals leads to PL emission peak steadily redshift from 650 nm (1.9 eV) to 780 nm (1.59 eV) (Supporting Information Figure S7). The excitation-dependent emission feature could be associated with the thickness distribution of Cu_3_VSe_4_ NSs and the effect of different surface functionalization on the NSs^40–43^. However, the emission peak at 690 nm has the highest intensity, suggesting that for most of the Cu_3_VSe_4_ NSs product, 460 nm is the optimal excitation wavelength, corresponding to a band gap of 1.80 eV.

To interrogate the effect of surface functionalization on the PL spectra of Cu_3_VSe_4_ NSs by conducting PL measurements on the Cu_3_VSe_4_ NSs that were subjected to ligand exchange. Although the same distribution of emission wavelengths remained, the main peak remained at 690 nm for the 460 nm excitation wavelength (Supporting Information Figure S3). The impact of thickness dependence of PL emission was documented for other materials, including layered 2D MoS_2_^44^ and WS_2_^45^. The FTIR spectra in Figure S4 (“Supporting Information”) demonstrated the complete removal of the organic ligands after the ligand exchange.

Theoretical predictions place sulvanites, including Cu_3_VSe_4_, in the optically active materials category, holding promise in applications including photodetectors and photovoltaics^19,21^. A photocurrent evaluation was carried out using Cu_3_VSe_4_ thin films obtained by depositing nanosheet dispersions (inks) on conductive, fluorine-doped tin oxide (FTO) substrates, in a photoelectrochemical setting. The fabricated Cu_3_VSe_4_ NSs-FTO thin film shows a homogeneous distribution of the nanosheets on the substrate and a uniform thickness of about 604 nm (Supplementary Figure S5). The Cu_3_VSe_4_ NSs thin films were further used to investigate the photoresponse of Cu_3_VSe_4_ NSs.

**Figure 8:**
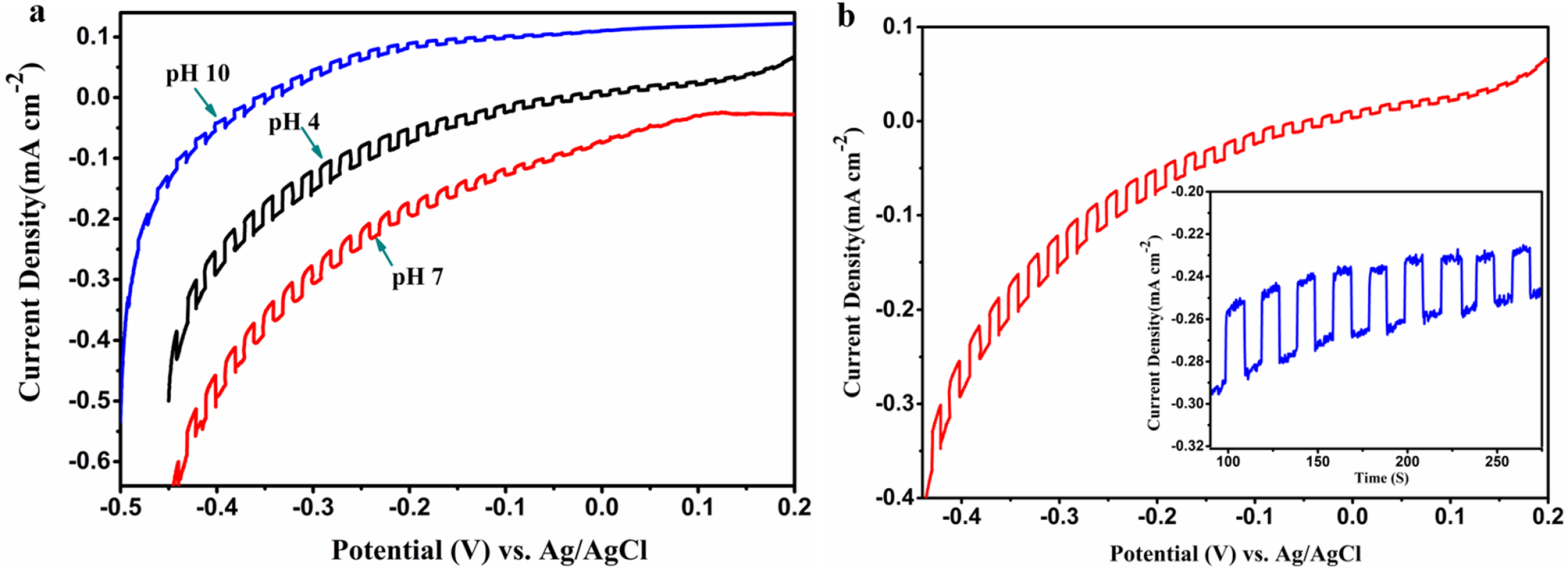
(**a**) Current–voltage (J–V) curve of the Cu_3_VSe_4_ NSs thin film in KCl aqueous solution of various pH values; (**b**) photocurrent response of the Cu_3_VSe_4_ NSs thin film in KCl aqueous solution with pH of 4 at − 425 mV.

Electrolyte pH affects the photoelectrochemical behavior of the semiconductor, impacting the rate of photo-reduction/photo-oxidation as well as the rate of electron–hole recombination in the system^46,47^. The current density–voltage (J–V) curves of Cu_3_VSe_4_ NSs were therefore explored, using a 0.6 M KCl aqueous solution with varying pH as electrolyte (Fig. 8a). The Cu_3_VSe_4_ NSs-FTO thin film exhibits a cathodic photocurrent response with highest photocurrent obtained in the electrolyte with pH 4, indicating that Cu_3_VSe_4_ is a p-type semiconductor. The cathodic photocurrent generation could be attributed to the H^+^ reduction^26^; Cu_3_VSe_4_ NSs illumination drives generation of electron/hole pairs, with the photogenerated electrons reaching the electrode/electrolyte interface reducing H^+^ to H_2_^32^. The effect of pH can be understood in terms of surface reaction on the Cu_3_VSe_4_ NSs-FTO thin film. At low pH, namely using acid KCl aqueous solution as the electrolyte, the proposed H^+^ reduction reaction at cathode (Cu_3_VSe_4_ NSs-FTO thin film) is $2{H}^{+}+2{e}^{-}\to {H}_{2}$, which consumes hydrogen ions; whereas, at high pH (pH = 10), the reaction at cathode consumes water rather than H^+^, being ${2H}_{2}O+2{e}^{-}\to {H}_{2}+{2OH}^{-}$ ^48,49^. The consumption of water takes place via an extra water dissociation ${H}_{2}O\leftrightarrow {H}^{+}+{OH}^{-}$, thus, the process of photocatalytic reduction is naturally slowed at higher pHs^50^, in turn, the larger amount of H^+^ present at acid pH facilitates the H_2_ production. Besides, at pH of 4, the surface of Cu_3_VSe_4_ NSs-FTO thin film is positively charged by adsorbing H^+^, facilitating the reduction reaction, whereas, at higher pH (pH of 7 and 10) the electrode/electrolyte interface is neutral or negatively charged, which eliminates the electrostatic interaction, or even creates a strong electrostatic repulsion towards Cl^−^ that results in a lower hole capture rate and in a low production of hydrogen^51^. The changing trend of photocurrent response is consistent with their photocatalytic H_2_ evolution. Therefore, the increase of photocurrent response at pH of 4 can be attributed to the larger capture of holes which reduces charge recombination, as well as the large amount of H^+^ which facilitating the H^+^ reduction reaction at the cathode/electrolyte interface. A chronoamperometry experiment using the same photoelectrochemical cell setup and the KCl aqueous solution with pH 4 as electrolyte was carried out at − 425 mV through several 10 s light on–off cycles (inset, Fig. 8b); a stable photocurrent of ~ 0.036 mA cm^−2^ was observed. According to literature, the bare FTO substrate sometimes presents photocurrent response during the electrochemical measurements^52–54^. A control experiment was performed with bare FTO as working electrode and the chronoamperometry experiment being conducted under the same condition with the measurement of Cu_3_VSe_4_ NSs-FTO thin film. The photocurrent response of bare FTO substrate is shown in Supplementary Fig S6 (red line), where no significant photocurrent is observed under the same conditions, suggesting that the photocurrent response of Cu_3_VSe_4_ NSs-FTO thin film is substantially resulted from the Cu_3_VSe_4_ NSs thin film. To investigate the stability of the photocurrent response of Cu_3_VSe_4_ NSs, a chronoamperometry measurement was conducted on the Cu_3_VSe_4_ NSs-FTO thin film which was kept in air for 6 months after the photoelectrochemical measurement. As shown in Figure S6 (“Supporting Information”) blue line, the Cu_3_VSe_4_ NSs-FTO thin film could still display active photoelectrochemical behaviour after 6 months. Furthermore, Cu_3_VSe_4_ NSs showed good stability in the electrochemical measurements, as the XRD pattern and TEM (Supporting Information Figure S7) of Cu_3_VSe_4_ NSs after the photoelectrochemical test indicate that the Cu_3_VSe_4_ preserved the nanosheet morphology and cubic crystals.

A comparative test was conducted using a thin film fabricated with Cu_3_VSe_4_ nanocrystals (NCs) on FTO as the photoelectrode (Supporting Information Figure S6). The observed photocurrent generated by Cu_3_VSe_4_ NSs-FTO thin film is around nine fold higher than the photocurrent produced by the reported Cu_3_VSe_4_ NCs-FTO thin film^26^. The photocurrent enhancement in Cu_3_VSe_4_ NSs could be ascribed to the higher surface area provided by the 2D geometry of the nanosheets.

**Figure 9:**
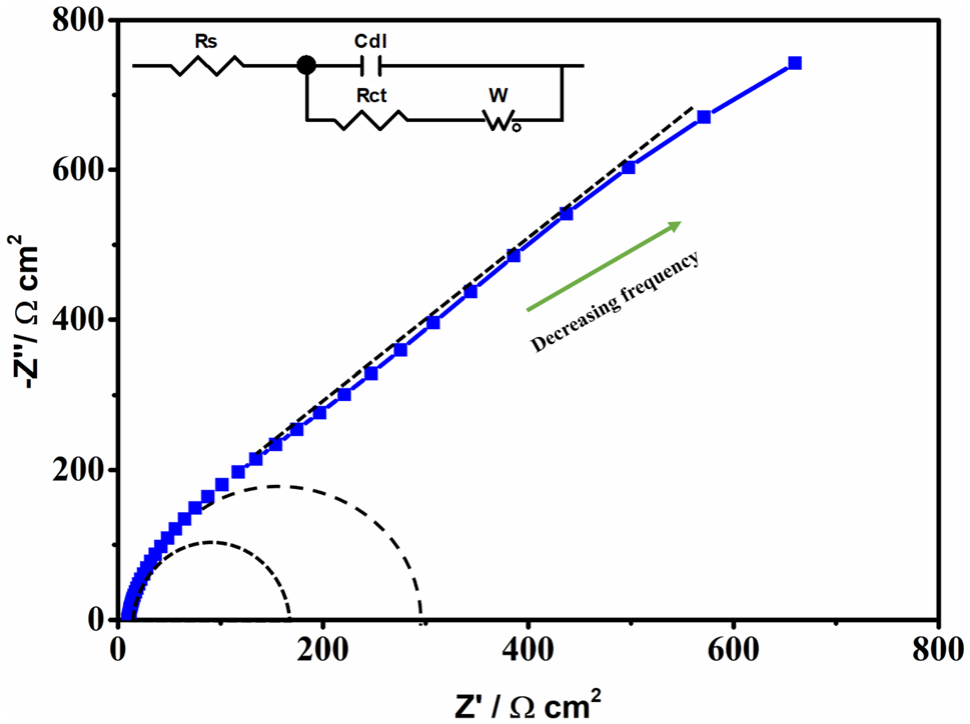
Nyquist plot of the prepared Cu_3_VSe_4_ NSs-FTO thin film measured in 0.6 M KCl aqueous solution with pH of 4 at the 0.215 V potential vs Ag/AgCl.

The electrochemical cell fabricated with the Cu_3_VSe_4_ NSs-FTO thin film was further subjected to an Electrochemical Impedance Spectroscopy (EIS) measurement to study the interfacial charge transfer characteristics at the semiconductor/electrolyte interface. Figure 9 shows the Nyquist plot obtained at the open-circuit potential of 0.215 V vs Ag/AgCl in the frequency range of 1 Hz to 100 KHz. An electrical equivalent circuit, as shown in the inset in Fig. 9, was designed to rationalize the charge-transfer and transport phenomena in the electrochemical cell, where R_s_, R_ct_, C_dl_, and W represents solution resistance, charge-transfer resistance, double-layer capacitance, and Warburg impedance, respectively. The first small semicircle at high frequency region is related to the solution resistance R_s_, whereas the large semicircle at low frequency region could be assigned to the charge-transfer resistance R_ct_ and the double-layer capacitance C_dl_ at the electrode/electrolyte interface^55,56^. Generally, the diameter of semicircle extrapolated in the Nyquist plot represents resistance R, in turn, reflects the reaction rate^57,58^. Herein, the charge transfer resistance (R_CT_) of Cu_3_VSe_4_ NSs-FTO thin film in 0.6 M KCl aqueous electrolyte was found to be 300 Ω cm^2^ from the second semicircle of the Nyquist plot. The linear part located at low frequency could be ascribed to the Warburg impedance W that corresponds to the diffusion processes.

## Conclusion

Quasi-2D Cu_3_VSe_4_ nanosheets with cubic crystal structure have been prepared through a templating method using binary VSe_2_ NSs as a solid-state precursor in a solution-phase approach, which enabled the preservation of the 2D morphology. Microscopy analyses (TEM, SEM, and AFM) highlighted the large lateral size and thin thickness of the synthesized Cu_3_VSe_4_ nanosheets. Three strong absorption bands at 382 nm, 552 nm, and 664 nm were observed for Cu_3_VSe_4_ nanosheets, indicating the presence of an intermediate bandgap. The emission peak redshift in the PL spectra, from 650 to 780 nm, when varying the excitation wavelength from 430 to 520 nm, is attributed to the size/thickness distribution and has been observed in other layered materials, including 2D MoS_2_^44^ and WSe_2_^45^. The mechanism study suggested that the formation of Cu_3_VSe_4_ nanosheets involves primarily the Cu_3_VSe_4_ nanosheets formation as a result of reaction of Cu(II) cations with VSe_2_ NSs. At the same time, unreacted Se present in the reaction reacts with Cu cations to form a minor Cu_2_Se is consumed during the reaction by reacting with VSe_2_ NSs and leads to Cu_3_VSe_4_ nanosheets.

To evaluate the conduction type of Cu_3_VSe_4_ NS, thin films were made by depositing the nanosheets (after ligand exchange) on FTO substrates. The fabricated Cu_3_VSe_4_ NSs thin film exhibited a p-type semiconductor behavior. A photocurrent of ~ 0.036 mA cm^−2^ was measured in an electrochemical setting, in a KCl aqueous solution at pH 4.0. The electrical impedance spectroscopy was measured assuming that the Cu3VSe4 NSs-FTO based electrochemical cell fits an equivalent circuit with the circuit elements of solution resistance (Rs), charge-transfer resistance (Rct), double-layer capacitance (Cdl), and Warburg impedance (W). The estimated charge transfer resistance value of 300 Ω cm^2^ obtained from the Nyquist plot provides an insight into the rate of charge transfer at the electrode/electrolyte interface.

The cascade synthesis method reported herein for the Cu_3_VSe_4_ ternary chalcogenides could be expanded to other significant chalcogenides that could benefit from 2D morphology. The lightweight nanostructures could be useful in a plethora of optoelectronic devices that support contemporary applications, including wearable electronics, biosensors and the Internet-of-Things (IoT).

## Methods

### Materials

All chemicals used in the experiment were used as received, without further purification. Vanadium (IV) oxide acetylacetonate (VO(acac)_2_, ≥ 98%) was ordered from Merck KGaA. Selenium powder (Se, 99.99%), oleylamine (OLA, 70%), 1-dodecanethiol (1-DDT, ≥ 98%), Copper(II) chloride dihydrate (CuCl_2_·2H_2_O, 99.999%), and formamide were bought from Sigma-Aldrich. Sodium sulfide (Na_2_S, anhydrous) was purchased from Alfa Aesar. ACS grade chloroform (CHCl_3_, ≥ 99.8%), toluene (C_7_H_8_, ≥ 99.5%), and methanol (CH_3_OH, 99.8%) were bought from Fisher Scientific. Ethanol (C_2_H_5_OH, 100%) was ordered from Decon laboratories. FTO Soda Lime glass substrates were purchased from MSE Supplies.

### Preparation of VSe_2_ nanosheets (NSs)

In the preparation of VSe_2_ NSs synthesis, Vanadium (IV) oxide acetylacetonate was used as the source of V^4+^ cations, whereas elemental Se served as the source of Se^2−^ anions. Vanadium (IV) oxide acetylacetonate (133 mg, 0.5 mmol) and 15 mL OLA were loaded in a 100 mL two-neck round bottom flask. The mixture was degassed for 30 min at 120 ℃ and flushed with argon. Meanwhile, Se powder (1 mmol, 78.96 mg) was mixed with 1 mL of 1-DDT and 3 mL of OLA in a 25 mL two-neck round bottom glass flask. The Se solution was stirred under vacuum at room temperature for 30 min and then purged with argon. Next, the temperature of vanadium precursor was raised to 140 ℃, followed by the swift injection of the Se solution. The reaction was further heated to 250 ℃ and maintained at this temperature for two hours to form the VSe_2_ nanosheets. Upon cooling, a mixture of OLA (9 mL) and I-DDT (1 mL) was added to remove the Se residues and the precipitated VSe_2_ nanosheets were collected by centrifugation. The nanosheets were subsequently washed twice with the same CHCl_3_ and C_2_H_5_OH mixture (V:V, 1:3). The final precipitates were collected and dried overnight in a vacuum oven.

### Preparation of Cu_3_VSe_4_ nanosheets

In a typical synthesis, the formed VSe_2_ OLA prepared as described above were not removed from suspension, and upon reaching two hours reaction time at 250 ℃ under argon atmosphere and a Cu (II) solution was rapidly injected. The Cu (II) solution was made by dissolving CuCl_2_·2H_2_O (136.4 mg, 0.8 mmol) in 5 mL of OLA. The reaction was kept at 250 ℃ for one hour. Afterward, the heating source was removed, and the reaction allowed to cool down to room temperature. Upon cooling, a mixture of CHCl_3_ and C_2_H_5_OH (V:V, 1:3) was added and the precipitated Cu_3_VSe_4_ nanosheets were collected by centrifugation. The product was further purified by washing twice with a mixture of CHCl_3_ and C_2_H_5_OH (V:V, 1:3). The precipitate was dried overnight in a vacuum oven.

### Mechanism study

To study the mechanism of Cu_3_VSe_4_ formation starting from the VSe_2_ nanosheets, the experiment described above was conducted with different reaction times from the point of addition of Cu(II) solution. The series included experiments with all parameters held the same and reaction times of 1minute, 5 min, 15 min, 30 min, 1 h, and 2 h.

### Ligand exchange with S^2−^

A ligand exchange process was conducted to replace the OLA coordinated to Cu_3_VSe_4_ nanosheet surface with S^2−^. In a typical experiment, Cu_3_VSe_4_ nanosheets were suspended in chloroform (8 mg mL^−1^) and 10 mL of the suspension was transferred to a 50 mL tube containing 10 mL of Na_2_S solution in formamide (0.2 M). Next, the mixture was vigorously shaken for 1 min and further allowed to rest until ligand exchange completion, when Cu_3_VSe_4_ NSs were fully transferred from the chloroform phase (lower) to the formamide phase (upper). Afterward, the clear chloroform phase was removed, and 5 mL distilled water and 20 mL of ethanol was added to the aqueous phase to precipitate the Cu_3_VSe_4_ NSs. The precipitate was purified by washing twice with a mixture of ethanol and distilled water (V:V, 4:1) followed by washing with a mixture of toluene and ethanol (V:V, 1:3). The resulting product was collected and dried in a vacuum oven overnight.

### Cu_3_VSe_4_ inks and thin film fabrication

The Cu_3_VSe_4_ NSs inks were prepared via dispersing 10 mg of ligand exchanged-Cu_3_VSe_4_ NSs into 1 mL of ethanol and ultrasonicating the dispersion for 10 min with a probe sonicator. The substrate used for ink deposition, 1″ × 1″ FTO-coated glass, were freshly cleaned using an ultrasonic bath using, in sequence: distilled water, methanol, and acetone, for 10 min each.

All Cu_3_VSe_4_ thin films used in this work were fabricated by bar-coating in ambient conditions. An amount of 20 μL of Cu_3_VSe_4_ ink was dispensed and coated on the FTO-coated glass. The film was dried for 1 min in air at 100 ℃, using a hot plate. The coating and drying process was repeated twice to complete the glass/FTO/Cu_3_VSe_4_ thin film.

### Photoelectrochemical measurements

To evaluate the photoelectrochemical performance, a three-electrode photoelectrochemical cell has been set up, consisting of an Ag/AgCl reference electrode, a platinum counter electrode, and the Cu_3_VSe_4_ NSs-FTO thin film as the photoelectrode. An aqueous solution of KCl (0.6 M) was used as the electrolyte. The pH of the KCl electrolyte was adjusted to either 4 or 10 using HCl or 2 M NaOH aqueous solution, respectively. The current density–voltage (J–V) dependence was investigated in the − 0.5 to 0 V range, with a cycle of 10-s light-off and 10-s light-on. The sweep rate was 2 mV s^−1^ and the LED light power was 2000 lumens. The photocurrent of the Cu_3_VSe_4_ NSs-FTO thin film using the same 10 s light-on/10 s light-off cycle recorded with a Pine Research Potentiostat.

### Characterization

The crystal structure and purity of prepared VSe_2_ and Cu_3_VSe_4_ nanosheets were determined by X-ray powder diffraction (XRD) using a Siemens Diffractometer D5000 (Cu Kα radiation, λ = 1.5405 Å). To confirm product purity by Raman spectroscopy, Raman spectra were obtained with a Renishaw Raman microscope equipped with a 633 nm laser. The morphology and size of the synthesized VSe_2_ and Cu_3_VSe_4_ were determined by transmission electron microscopy (TEM) imaging, using a Philips CM200, and scanning electron microscopy (SEM) imaging, with a JEOL 6330F. Elemental distribution of the Cu_3_VSe_4_ nanosheets was determined with the energy dispersive spectroscopy (EDS) feature of the JEOL 6330F SEM. The oxidation states of the Cu, V, and Se elements of the synthesized Cu_3_VSe_4_ nanosheets were confirmed via X-ray photoelectron spectroscopy (XPS) in a VG Escalab 220i-XL equipped with an Al Kα source. Photoluminescence (PL) measurements of Cu_3_VSe_4_ nanosheets were carried out with a Perkin Elmer LS-55 Luminescence Spectrometer. The absorption spectrum of Cu_3_VSe_4_ nanosheets was collected using an Agilent Cary 5000 UV–Vis–NIR spectrophotometer. The thermal stability of the Cu_3_VSe_4_ nanosheets was determined by thermogravimetric analysis (TGA) using a TA Instrument SDT-Q600 Simultaneous TGA/DSC. The WaveNow Potentiostat (PINE research) was used to determine the photoelectrochemical behavior of the Cu_3_VSe_4_ NSs-FTO thin film.

## Supplementary Information

Supplementary Figures.

## Data Availability

All data generated or analyzed during this study are included in this article (and its “Supporting Information” file).
