# Supplementary material for: Cascade synthesis and optoelectronic applications of intermediate bandgap Cu3VSe4 nanosheets
Source: Sci Rep. 2020 Dec 10;10:21679. doi: 10.1038/s41598-020-78649-9 (PMC8097018; doi:10.1038/s41598-020-78649-9)
Supplement: Supplementary file 1 — Supplementary Figures. [file 41598_2020_78649_MOESM1_ESM.docx]

**Supporting Information**

**Cascade Synthesis and Optoelectronic Applications of Intermediate Bandgap Cu_3_VSe_4_ Nanosheets**

Mimi Liu^1^ , Cheng-Yu Lai^1^, Meng Zhang^2^, and Daniela R Radu^1,3, *^

^1^Department of Mechanical and Materials Engineering, Florida International University, College of Engineering and Computing, Miami, 33174, United States of America
^2^Georgia Institute of Technology, School of Materials Science and Engineering, Atlanta, 30332, United States of America

^3^Department of Materials Science and Engineering, University of Delaware, Newark, Delaware, United States of America
^*^corresponding. [dradu@fiu.edu](mailto:dradu@fiu.edu)

**Supplementary Information**


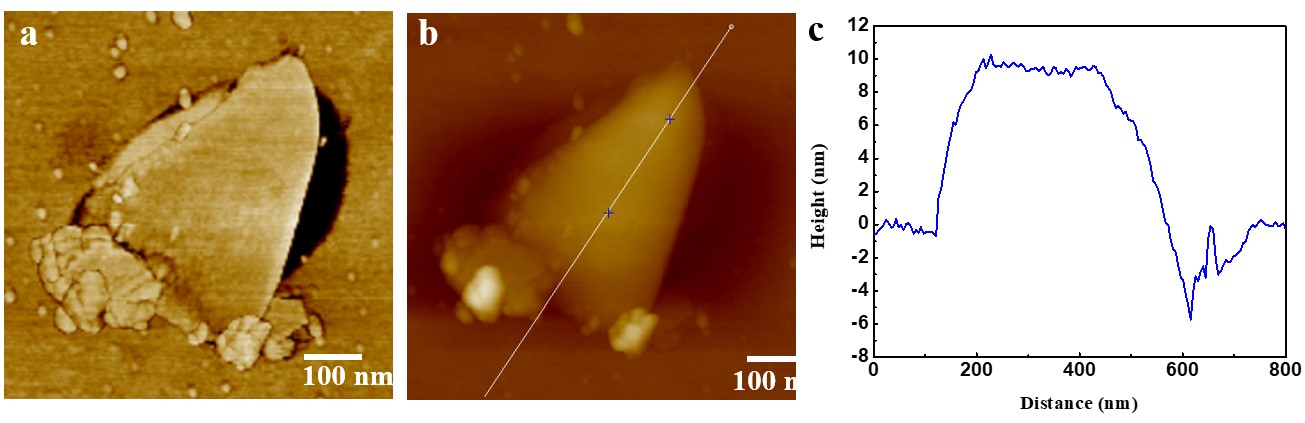


**Figure** **S1** (a) AFM image. (b)Topography of the synthesized Cu_3_VSe_4_ nanosheets. (c) Height profiles measured along the white line in panel (b).


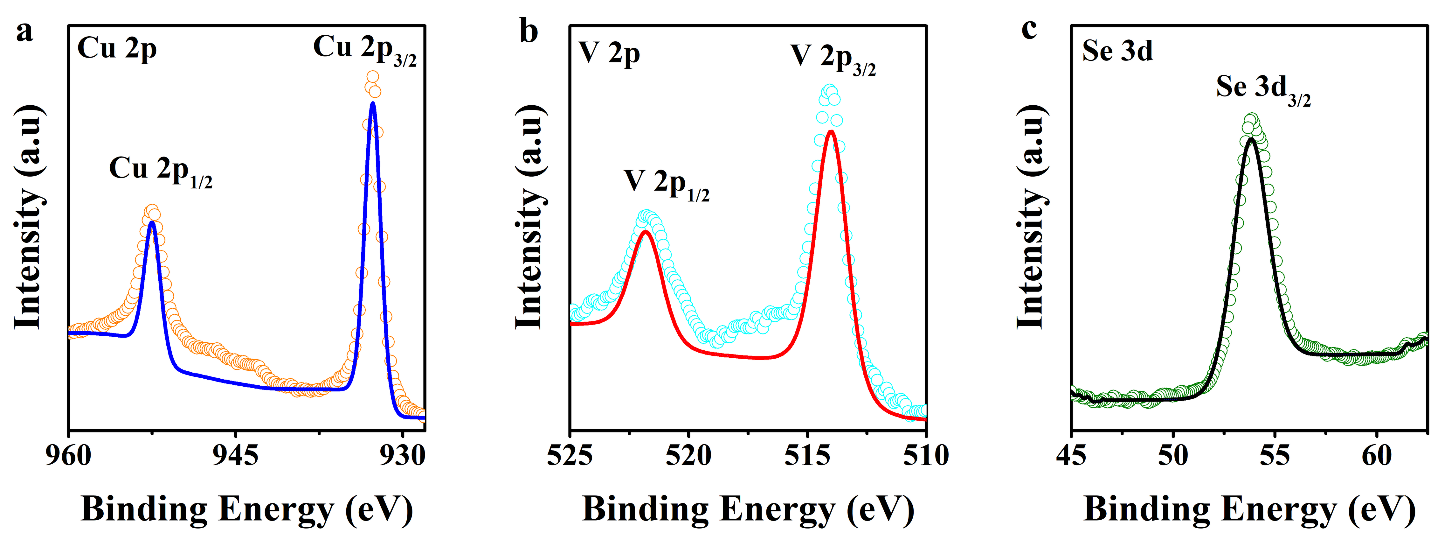


**Figure S2** XPS spectra of the Cu 2p, V 2p and Se 3d peaks of synthesized Cu_3_VSe_4_ nanosheets.

X-Ray Photoelectron Spectroscopy (XPS) measurements were carried out to gain insight into the chemical and electronic structures of the synthesized Cu_3_VSe_4_ nanosheets. Fig S2 displays the XPS spectra of the Cu 2p, V 2p, and Se 3d orbitals of the synthesized Cu_3_VSe_4_ nanosheets. Two distinct binding energies of Cu, at 932.6 eV and 952.5 eV, correspond to the Cu 2p_3/2_ and Cu 2p_1/2_, respectively, indicating the presence of Cu^+^ ( Fig S2a**)**.^1-3^ Fig S2b presents the binding energies of V 2p, where two characteristic peaks located at 514.1 eV (V 2p_3/2_) and 521.8 eV (V 2p_1/2_) with a separation of 7.7 eV. The peak at 514.1 eV is associated with the binding energy of V 2p_3/2_  for V^3+^ ions.^4,5^ However, considering that Cu_3_VSe_4_ is the only phase of Cu_3_VSe_4_ nanosheets in the XRD pattern, the oxidation state of the vanadium element is expected to be V^5+^. When compared to the Cu_3_VSe_4_ nanocrystals we recently reported^6^, the binding energy of V^5+^ exhibits a negative shift of 2.6 eV.

A similar behavior was reported for the sulfur counterpart, Cu_3_VS_4_ nanocrystals.^7^ The report stated that the V oxidation state in Cu_3_VS_4_ is indeed +5, and attributed a similar shift of 2.9 eV to a potential V(IV)-S, due to the presence of S^-2^, given that V(IV)-O occurs at 517.2 eV. Besides, interesting example of chemical environment-dependent core-level energies shift were reported for the carbon atom, where the binding energy of carbon atom shifts to a more negative region when bonding to sulfur.^8^ The synthesized Cu_3_VSe_4_ NSs capped with oleylamine or 1-dodecanethiol through the bonds between S atom of 1-DDT and the cations of Cu_3_VSe_4_ NSs or the bonds between the N atom of OLA to the cations of Cu_3_VSe_4_ NSs. Based on literature, binding energies of V^5+^ shift to the more negative region could be related to the chemical environment. However, further characterization is required to demonstrate the explanations.

As shown in Fig S2C, the Se 3d_3/2_ was fitted with a peak at 53.8 eV which corresponded to the Se 3d_3/2_ of Se^-2^. ^9-11^


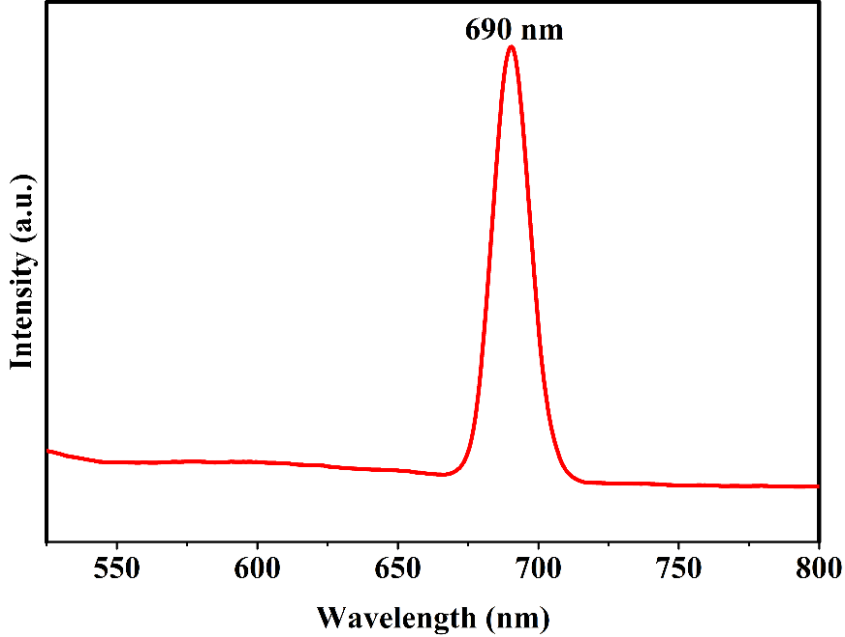


**Figure S3** Photoluminescence (PL) spectra of ligand exchanged Cu_3_VSe_4_ NSs.


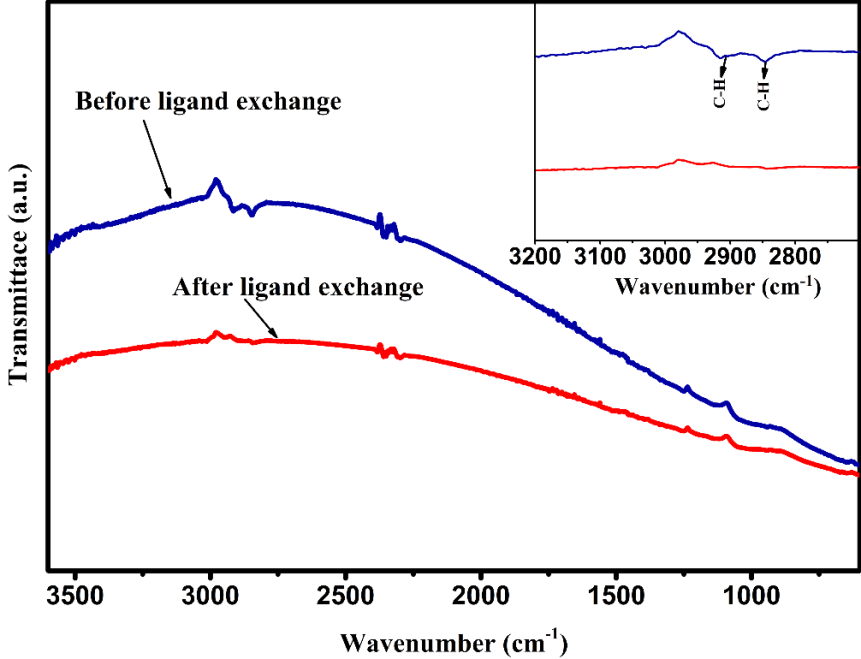


**Figure S4** FTIR spectra of the as-synthesized Cu3VSe4 NSs ( blue line) and Cu3VSe4 NSs with ligand exchange (red line).

Organic ligand presence on the Cu_3_VSe_4_ NSs could impede the charge transport among the nanomaterials and in turn, reducing the conductivity of the fabricated thin film. To improve the charge transfer among Cu_3_VSe_4_ NSs, it is essential to remove the organic ligands of the Cu_3_VSe_4_ NSs. Thus, a ligand exchange process was carried out to replace the organic ligands on the surface of Cu_3_VSe_4_ NSs with inorganic ligand S^2-^. The top trace (blue) in Fig S4 displays the FTIR spectrum of the as-synthesized Cu_3_VSe_4_ NSs, where two prominent characteristic bands located at around 2852 cm^-1^ and 2925 cm^-1^ corresponding to C-H stretching, associated to the OLA presence on Cu_3_VSe_4_ NSs. No band corresponding to 1-DDT at 2563 cm^-1^ was observed, suggesting that this ligand does coordinate to the surface of the nanosheets. ^12,13^ The disappearance of the C-H stretching (Fig S4, bottom trace**,** red) in the FTIR of the treated material indicates complete removal of the organic ligand.


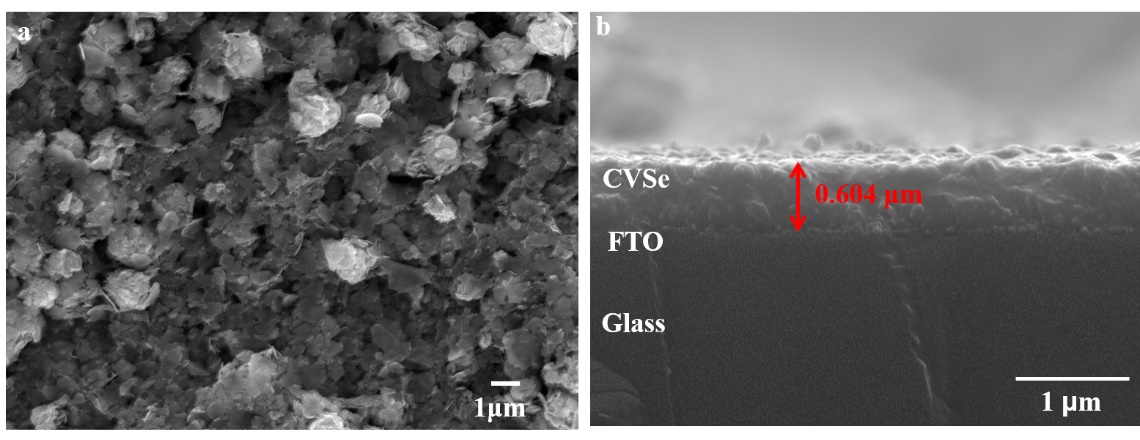


**Figure S5** (a) SEM image of the surface. (b) cross section of the Cu_3_VSe_4_ NSs-FTO thin film.


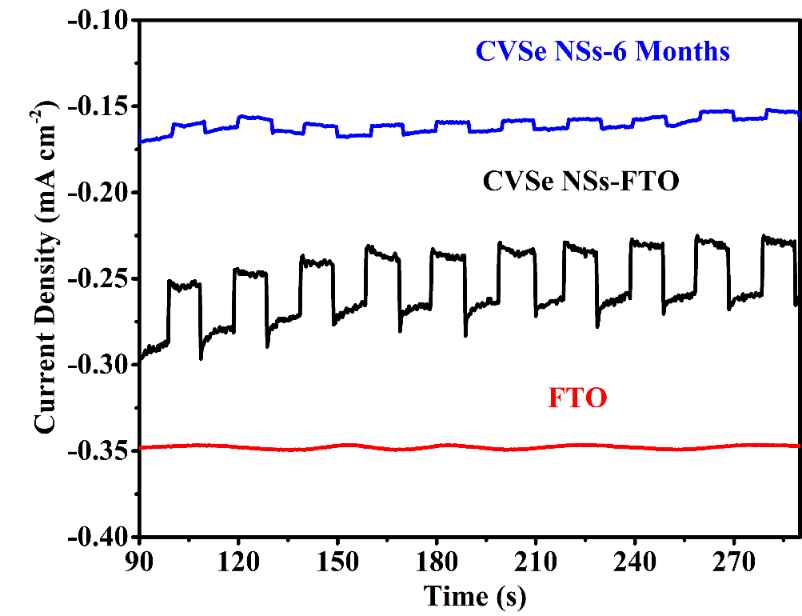


**Figure S6** Photocurrent response of FTO, CVSe NSs-FTO, CVSe NSs-FTO-6 Months.


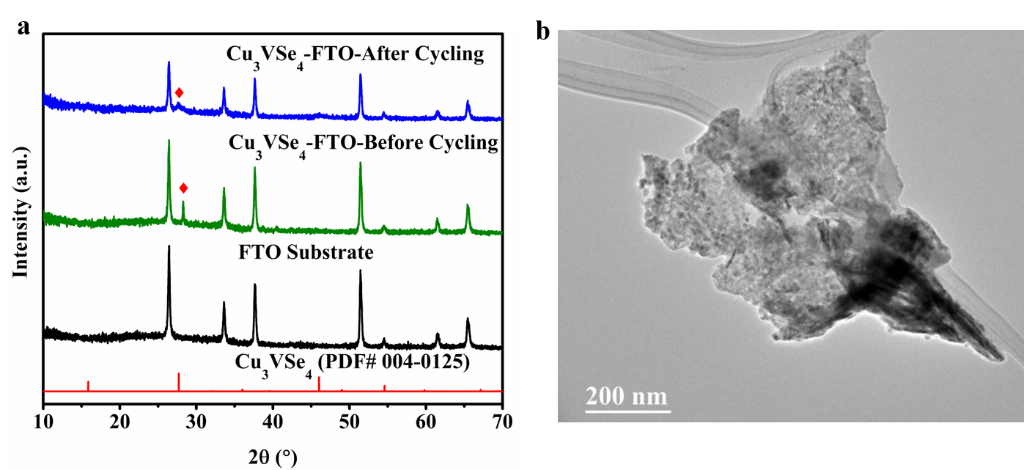


**Figure S7** XRD pattern and TEM image of Cu_3_VSe_4_ NSs after the photoelectrochemical test.


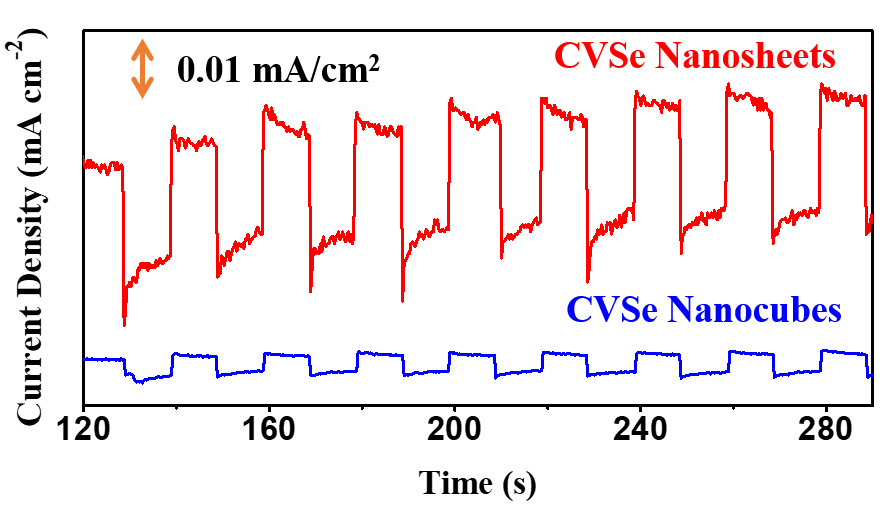


**Figure S8** Comparison of photocurrent of Cu_3_VSe_4_ NSs-FTO thin film and Cu_3_VSe_4_ NCs-FTO thin film.

**
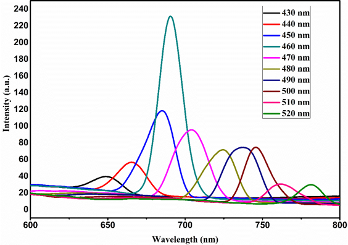
**

**Figure S9** The photoluminescence (PL) spectra of synthesized Cu_3_VSe_4_ NSs using different excitation wavelengths.

**References.**

1 Mohammadnezhad, M. *et al.* Synthesis of highly efficient Cu2ZnSnSxSe4−x (CZTSSe) nanosheet electrocatalyst for dye-sensitized solar cells. *Electrochimica Acta* **340**, doi:10.1016/j.electacta.2020.135954 (2020).

2 Kim, M.-w. *et al.* Electrosprayed copper hexaoxodivanadate (CuV2O6) and pyrovanadate (Cu2V2O7) photoanodes for efficient solar water splitting. *Journal of Alloys and Compounds* **708**, 444-450, doi:10.1016/j.jallcom.2017.02.302 (2017).

3 Zhang, X. *et al.* Simple one-pot synthesis of Cu4SnS4 nanoplates and temperature-induced phase transformation mechanism. *CrystEngComm* **22**, 1220-1229, doi:10.1039/C9CE01772K (2020).

4 Courcot, D. *et al.* Effect of the sequence of potassium introduction to V2O5/TiO2 catalysts on their physicochemical properties and catalytic performance in oxidative dehydrogenation of propane. *Catalysis Today* **33**, 109-118, doi:<https://doi.org/10.1016/S0920-5861(96)00098-3> (1997).

5 Laurenti, M. *et al.* Lead-free piezoelectrics: V(3+) to V(5+) ion conversion promoting the performances of V-doped Zinc Oxide. *Sci Rep* **7**, 41957-41957, doi:10.1038/srep41957 (2017).

6 Liu, M., Lai, C.-Y., Selopal, G. S. & Radu, D. R. Synthesis and optoelectronic properties of Cu3VSe4 nanocrystals. *PLOS ONE* **15**, e0232184, doi:10.1371/journal.pone.0232184 (2020).

7 Mantella, V. *et al.* Synthesis and Size-Dependent Optical Properties of Intermediate Band Gap Cu3VS4 Nanocrystals. *Chemistry of Materials* **31**, 532-540, doi:10.1021/acs.chemmater.8b04610 (2019).

8 Taucher, T. C., Hehn, I., Hofmann, O. T., Zharnikov, M. & Zojer, E. Understanding Chemical versus Electrostatic Shifts in X-ray Photoelectron Spectra of Organic Self-Assembled Monolayers. *J Phys Chem C Nanomater Interfaces* **120**, 3428-3437, doi:10.1021/acs.jpcc.5b12387 (2016).

9 Bernede, J. C., Hamdadou, N. & Khelil, A. X-ray photoelectron spectroscopy study of CuFeSe2 thin films. *Journal of Electron Spectroscopy and Related Phenomena* **141**, 61-66, doi:10.1016/j.elspec.2004.07.003 (2004).

10 Chen, G. *et al.* Low cost preparation of Cu2ZnSnS4 and Cu2ZnSn(SxSe1−x)4 from binary sulfide nanoparticles for solar cell application. *Journal of Power Sources* **262**, 201-206, doi:10.1016/j.jpowsour.2014.03.075 (2014).

11 Zhou, X. *et al.* Amorphous, Crystalline and Crystalline/Amorphous Selenium Nanowires and Their Different (De)Lithiation Mechanisms. *Chemistry of Materials* **27**, 6730-6736, doi:10.1021/acs.chemmater.5b02753 (2015).

12 Feizi, S., Zare, H. & Hoseinpour, M. Investigation of dosimetric characteristics of a core–shell quantum dots nano composite (CdTe/CdS/PMMA): fabrication of a new gamma sensor. *Applied Physics A* **124**, doi:10.1007/s00339-018-1837-5 (2018).

13 Zhang, B. Q. *et al.* Colloidal Synthesis and Thermoelectric Properties of CuFeSe(2) Nanocrystals. *Nanomaterials (Basel)* **8**, doi:10.3390/nano8010008 (2017).
